# Citizen science data suggest that a novel rig improves landing rate and reduces injury and handling time in recreational angling with artificial lures in Baltic pike (*Esox lucius*)

**DOI:** 10.7717/peerj.4744

**Published:** 2018-05-17

**Authors:** Jens Jakob Bursell, Robert Arlinghaus

**Affiliations:** 1 Outdoor Consult, Lynge, Denmark; 2 Integrative Fisheries Management at the Faculty of Life Sciences, Leibniz-Institute of Freshwater Ecology and Inland Fisheries, Berlin, Germany

**Keywords:** Catch-and-release, Landing rate, Bleeding, Hook wound, Release-rig, Unhooking time, Catch rate, Gear effectiveness, Pike

## Abstract

The optimal terminal gear in hook-and-line recreational fishing maximizes landing rates and minimizes injury to the fish because some fish will be released after capture. We designed a novel rig configuration in artificial lure fishing for top predators and examined its effectiveness in angling for Baltic northern pike (*Esox lucius*) using a citizen science approach based on observational data collected from volunteer anglers in the field. The novel rig included two changes to traditional rig designs common to artificial lure angling. First, hooks were mounted in a way giving better hook exposure and eliminating lever-arm effects from the lure to the hooks once a fish is hooked. This construction allowed the second change, being a shift to hooks 4–5 sizes smaller than those used on traditional hook mounts. We analysed observational data collected by volunteer anglers using either the novel rig or a standard rig mount in two types of artificial lures (softbait and hardbait) of the same size (about 17 cm). Using *N* = 768 pike contacts as input data, we showed the landing rates of pike targeted with artificial lures significantly and substantially increased from 45% with normal-rigs to 85% when the same lure types were fished with the novel rig configuration. Lure type and water temperature had no effects on landing rates. Moreover, hardbaits on normal-rigs produced significantly more injury, bleeding and elevated unhooking time compared to fish captured on hardbaits with release-rigs. We conclude that simple changes to traditional hook sizes and mounts in lure fishing may benefit both anglers and the fishes that are to be released and that citizen science projects with volunteer anglers are able to provide good data in proof-of-concept studies. Further experimental studies are needed to differentiate hook size from hook mount effects because both variables were confounded in the results of the observational data presented here.

## Introduction

Catch-and-release (C&R) is common in recreational angling. It is conducted either mandatorily in response to harvest regulations or voluntarily (Arlinghaus et al., 2007a). Under open access situations with unlimited angling effort, C&R fishing will contribute to a sustainable fishery only if the hooking mortality is low (Coggins Jr et al., 2007; Johnston, Beardmore & Arlinghaus, 2015). In addition, it is desirable to minimize the sublethal effects of C&R resulting from injury and acute stress (Brownscombe et al., 2017; EIFAC, 2008) because these impacts can lead to behavioral impairments (which can facilitate post-release predation, Raby et al., 2013), foster disease outbreaks (sometimes in a delayed fashion, Steeger et al., 1994; Margenau, 2007) and contribute to impaired growth and reproduction Arlinghaus et al., 2007a; Arlinghaus et al., 2007b; Cooke & Sneddon, 2007; Klefoth, Kobler & Arlinghaus, 2011; Meka, 2004; Richard et al., 2013). Using gear and handling techniques that minimize stress and injury caused by catching and landing as well as C&R are also an important ethical considerations (Arlinghaus et al., 2007b).

Deep hooking and the amount of injury induced by hooking and hook removal are amongst the most important factors affecting mortality in C&R across a range of species (Bartholomew & Bohnsack, 2005; Hühn & Arlinghaus, 2011; Margenau, 2007; Munoeke & Childress, 1994). Hooking the fish deeply can be avoided by using large hooks/baits/lures (Alós et al., 2008; Arlinghaus et al., 2008a; Wilde, Pope & Durham, 2003), artificial lures rather than natural bait (Arlinghaus et al., 2008a; Grixti, Conron & Jones, 2007), using fixed weights in fishing with natural baits for bottom-feeding fish (Beckwith Jr & Rand, 2005; Rapp, Cooke & Arlinghaus, 2008) or choosing particular bait/lure types (Alós et al., 2009; Stålhammer et al., 2014; Wilde, Pope & Durham, 2003). Also the hook size and type of mounting of hooks on the rig can affect the injury induced by recreational angling gear and its landing rate (Alós et al., 2008; Rapp, Cooke & Arlinghaus, 2008). New rig innovations are needed that maximize landing rates while minimizing injury in top predatory fishing, similar to the innovation caused by the bolt-rig in angling for large benthivorous fish (Rapp, Cooke & Arlinghaus, 2008).

If new tackle innovations, mounts and rigs are to be accepted by anglers they must reduce injury and post-release mortality without penalizing landing and catch rates (Alós et al., 2008; Schaeffer & Hoffmann, 2002). For example, barbless hooks on natural bait are known to reduce catch rates (Alós et al., 2008), which is why this hook type may not be universally accepted by anglers. In angling for carp (*Cyprinus carpio*), small barbed hooks on bolt-rigs have shown to increase landing and catch rates and at the same time inflict less injury to the fish compared to larger hooks of the same pattern (Rapp, Cooke & Arlinghaus, 2008). Our aim was to develop and test a rig having similar outcomes in recreational angling for top predators with artificial lures. We chose a citizen science approach where data was collected by volunteer anglers and interpreted the observational data presented here cautiously as suggesting treatment effects. However, we consider the data strong enough to serve as a proof-of-concept.

In the northern hemisphere, the northern pike (hereafter called pike) is a highly desired recreational target in many freshwaters and low salinity coastal zones (Arlinghaus, Bork & Fladung, 2008; Arlinghaus et al., 2018). C&R—mandatory and voluntary—is common in recreational fishing for pike (Arlinghaus et al., 2009; Crane et al., 2015). Hooking mortality in pike increases, as in many other species, with deep hooking, injury and bleeding (Arlinghaus et al., 2008a). Arlinghaus et al. (2008a) showed that hooking in the gills increases unhooking time, which elevates air exposure and may cause stress (Arlinghaus et al., 2009). However, deep hooking does not necessarily mean the pike bleeds, and pike that are bleeding do not necessarily die (Arlinghaus et al., 2008a; DuBois et al., 1994). Deep hooking can be reduced by avoiding small lures (Arlinghaus et al., 2008a) and certain types of baits, such as natural bait (Arlinghaus et al., 2008a) or spinfly (Stålhammer et al., 2014). Additionally, the environment plays a role in deep hooking. In Baltic pike, Stålhammer et al. (2014) showed that low temperatures skewed hooking locations towards deeper hooking positions, but the underlying mechanism remained unclear. It is important to further study factors that help avoiding deep hooking and help minimize unnecessary damage to the fish, while at the same time maintaining or even increasing catch and landing rates to facilitate implementation in practice among anglers. To that end, we developed a new type of rig for pike recreational angling with artificial lures, the so-called release rig (see submitted video here: https://figshare.com/articles/_/5833803). The main purpose of the rig innovation we present is to create a new system allowing the use of much smaller and thus likely better penetrating hooks, thereby supposedly maximizing landing rates and minimizing damage to the fish.

To examine whether the above assumption holds true, we designed a citizen science study where volunteer anglers fished the release-rig with small hooks and a normal rig mount with large hooks on two types of similar-sized artificial lures over the course of several years. Here, we present the collected observational data which is able to measure the combined effect of two changes (hook size and hook mount) represented in the release rig compared to angling outcomes of a traditional hook mount/size as represented in a standard lure (video) while controlling for lure type and lure size, on landing rates and injury when angling for Baltic pike. It was assumed that the combined effect of two modifications of a traditional rig in lure angling for pike would enable the angler to exploit the full potential of small hooks in terms of better penetration and hook hold while inducing less damage to the fish (Rapp, Cooke & Arlinghaus, 2008). We used observational data to test this hypothesis and consider the present study a proof-of-concept suggestive of the documented effects. We reserve further work to disentangle the relative contributions of hook mount and hook size on angling outcomes in lure angling for pike in a strict experimental setting. The principles behind the release-rig are not confined to pike. But here we focus on pike as a suitable model of a top predator targeted with artificial lures.

## Methods

### Description of the release-rig

**Figure 1:**
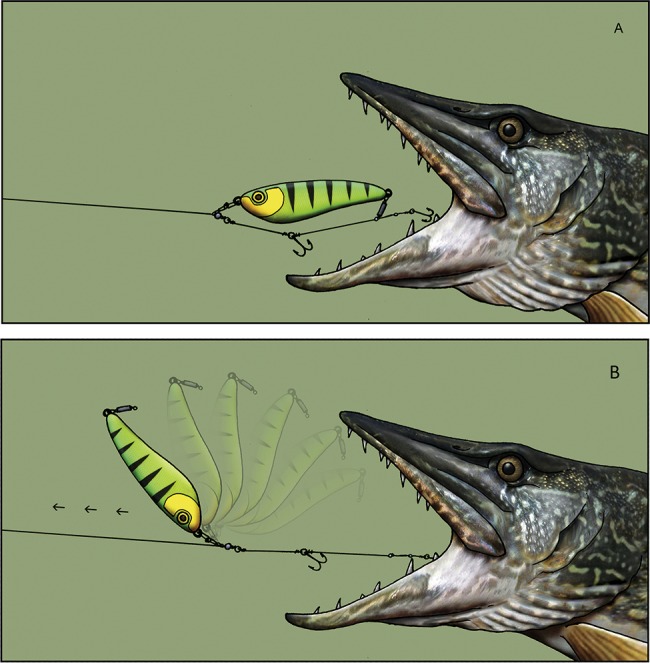
Visualization of the functionality of the release rig. (A) During cast and retrieve the hooklink is semi-fixed to the lure, and (B) the first time the fish bites over the lure and shakes its head after the bite, the lure is instantly released from the hook link (hence the name of the release-rig), so it can slide up the trace. Illustration: Carsten Madsen—http://www.underground-illustration.dk.

**Figure 2:**
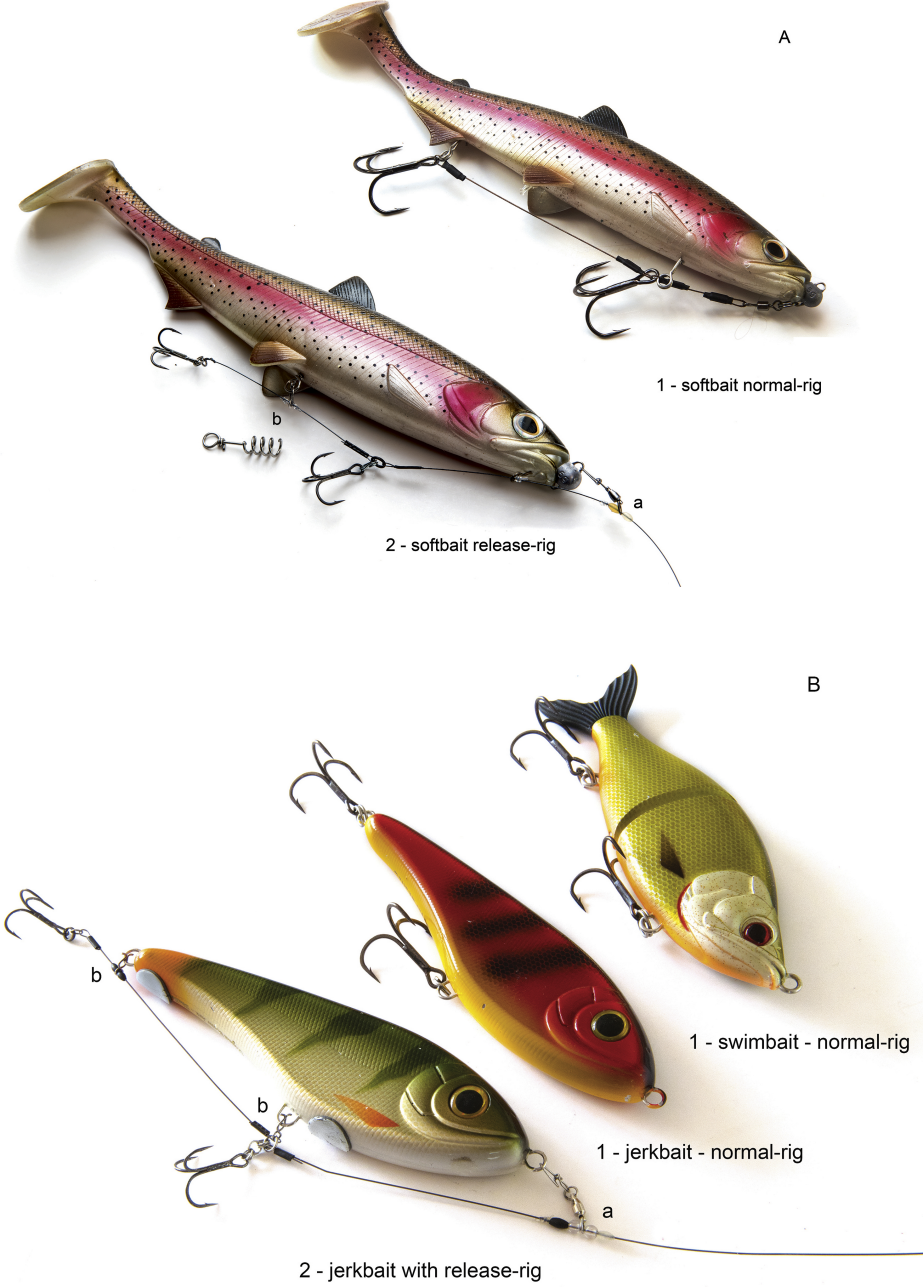
Lures and rigs used in the study. (A) Example of softbait (shad) with normal-rig (1) and with release-rig (2). Note the smaller hook-sizes in the release-rig. (B) Example of hardbaits (jerkbait and swimbait) with normal-rig (1) and with release-rig (2). Note the smaller hook sizes in the release-rig. Photo: Jens Bursell—http://www.bursell.dk.

**Figure 3:**
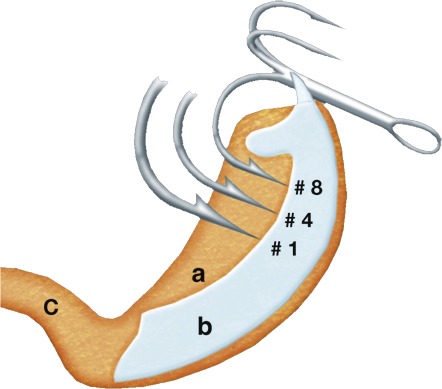
Schematic drawing based on X-ray photo from cross-section of pike in the 70–90 cm range. Small hooks from size 4 and down will often be able to get a grip with the barb in the soft tissue (a) over the hard and virtually impenetrable jawbones (b). Very big hooks often get a grip around the jawbone and down through the soft mouth floor (c). On hooks over size 2 the barb will rarely get a grip in the soft tissue (a) over the jawbone. Illustration: Carsten Madsen—http://www.underground-illustration.dk.

The bony jaws of the pike mean that the species can be difficult to hook, and consequently, many fish are either not hooked or lost during the fight (Arlinghaus et al., 2008b; Pullen et al., 2017). The original release-rig for pike was designed in 2007–2008 and first published in the popular angling media (Bursell, 2009). The release-rig we tested constituted an improved version of the first prototype rig. The final version of the release-rig constitutes a modification of traditional hook mounts in lure fishing in two key areas. First, by changing the way the hooks are attached to the artificial lure, they are detached from the lure after a bite, hence the term “release-rig” (Fig. 1). This was meant to reduce a lever-arm effect from the lure to the hooks, thereby allowing the second modification—to drop down 4–5 hook sizes compared to the hook sizes on a standard lure type of identical size (Fig. 2). Large hooks need more power to penetrate (see video) and hence may reduce landing rates in pike as in other species (Rapp, Cooke & Arlinghaus, 2008). We assumed that by avoiding a lever-arm effect, the release-rig would allow smaller hooks to be used. The shorter hook points (measured as distance from point to backside of barb) of small hooks means they can more easily achieve a hook hold with the barb sinking into the thin soft layer of tissue on top of the hard bony jaws in the mouth of the pike compared to large hooks (Fig. 3). However, as mentioned above, to allow the use of reduced hook sizes on traditional artificial lures for pike fishing it was necessary to alter the hook mounts to allow the small hooks to get detached from the artificial lure during the fight (Fig. 1). Otherwise, the leverage from the lure on the hooks could multiply the pressure on the hooks, potentially causing the smaller and thinner hooks to collapse, break or tear such a big wound that the hook would fall out, especially when fighting large specimens (see video).

In light of the above, the detailed configuration of the final release-rig version tested in the present paper was different from the normal rig mount in lure fishing in various ways. On the release-rig, the front of the lure is fixed to the rig via a cross lock swivel (a) (Fig. 2). In the middle and/or rear end, the hook link is semi-fixed via a “release-clip” (b) (Fig. 2), which ensures that the hooks hang in the right position during cast and retrieve. When a fish strikes the lure, the hook link with the release-clip is shaken off (Fig. 1B) ensuring no leverage from the stiff lure on the hooks, thereby assuming to aid retention of the fish. Additionally, when a heavy lure is slung around during the fight, this extra force on the hook may enlarge the hook wound and create a higher risk of the hook falling out. This effect is counteracted on the release-rig because the lure can slide up the trace during the fight (Fig. 1B). More details on how the release-rig is constructed and works for pike is shown in Bursell (2017) and on the video in https://figshare.com/articles/_/5833803. Following development of the rig over the course of several years, we used the final version of the release-rig with small hooks in this study by conducting an observational analysis on its functionality relative to the same lure types and sizes fished with normal rigs and large hooks using volunteer, avid pike anglers known in person to the first author. All anglers had a national fishing license and collected the data during their normal angling activities as covered by the Danish fishing law and Danish legislation for animal experimentation.

### Study site and design

The study was conducted in Baltic pike fished around the Danish and German coastline in the same salinity zone using casting techniques. Two lure types of the same approximate size commonly used by Baltic pike anglers were used—softbaits (Fig. 2A) and hardbaits (Fig. 2B). In pike, softbaits are known to increase catch rates relative to spoons (Arlinghaus et al., 2017) and are thus increasingly popular among anglers. Hardbaits are a traditional lure used in predator fishing. We standardized the lure size of the two lure types we used as the lure size is known to affect deep hooking (Arlinghaus et al., 2008a). We chose lengths of 17 ± 3 cm, which is a known size that increases the likelihood of catching mainly legal-sized pike (Arlinghaus et al., 2008a)—and strongly reduces to almost zero the bycatch from perch (*Perca fluviatilis*) and seatrout (*Salmo trutta*) (Arlinghaus et al., 2008a; Arlinghaus et al., 2017). We used the same lure types but rigged either with a normal-mount with large hooks (henceforth normal-rig) (1) or a release-rig with small hooks (2) (Fig. 2). This design means we can only study combined effects and cannot differentiate among the relative contributions of the hook mount or the size of hooks in our study.

Both lure types were mounted with two trebles of the same model (Owner ST 36 BC X) (Fig. 2). With lures fished the traditional way (normal-rig), industrial standard size hooks were used (“large hooks”, size 1/0–2/0), and hooks 4–5 sizes smaller (“small hooks”, size 6–4) were mounted on the release-rigs. Lures fished with release-rigs were added extra weight on the belly as compensation for the smaller and lighter hooks. The same standard medium-heavy pike equipment was used for traditional methods vs. release-rigs: rods with casting weights 50–120 g, braided mainline and multiplier/baitcasters. A contact or bite was defined as attack on the lure resulting in a distinct pull in the line that could be felt in the rod—including detected but unhooked bites lasting just a fraction of a second. All volunteer anglers taking part in the study were recruited by the first author and instructed. They were all very versatile and experienced anglers that can well differentiate a bite from just hitting a stone or snag. Bites seen visually but not transmitted through line were not counted. By-catch of other fish species with these big lures is highly unlikely in the shallow areas of the Baltic, and thus all contacts were counted as pike contacts.

The data collection was registered by four experienced anglers including the first author serving as citizen scientists fishing low salinity brackish waters in the Baltic Sea in the years 2013 to 2017. The data was collected during private leisure fishing and thus represents observational data by citizen scientists. All anglers were highly avid pike anglers. They were carefully instructed by the first author to note down fish contacts and landed pike whenever fishing with either of the two lure types of the prescribed size—or when fishing in the same boat, they told the first author about all contacts for him to note down. Anglers were free to choose which of the two lure types to use, and the anglers were also free to choose the colour. Given the anglers some freedom to choose among the timing of the two lure types and the colours they wanted to use was important to incentivize cooperation. Note that previous research in the visual predator largemouth bass (*Micropterus salmoides*) did not find any effect of colour of lures on catch rates (Moraga, Wilson & Cooke, 2015), and we assumed the same for our study. We first collected landing rate data for both lure types with the normal-rig during the years 2013–2015. From 2015–2017, data on the release-rig was collected. This sequence effect was caused by parallel initiatives in developing the final version of the release-rig, which took several years to complete. We assumed the basic fishing conditions to be equivalent in these two study periods as we were unable to statistically control for potential annual effects. Anglers were the same in all study years and thus a skill effect was controlled. All pike contacts and landed fish were registered, and it was noted if the contacts happened in warm water in the summer half year (May–October) or in cold water in the winter half year (November–March). Size of fish landed was however not recorded in order to minimize damage to the fish, burden on the anglers and due to concerns in comparability of the measurements. Also it is known in pike fishing that lure size determines pike size (Arlinghaus et al., 2008a) and lure size was controlled. Fishing was done through all seasons except periods where the water was iced over or the pike were protected (April, 1 to May, 15 of each study year). The full study consisted of two summer plus two winter seasons for each rig type. Overall, we analysed 768 pike contacts for the effect of release-rig relative to standard rig, lure type and water temperature (cool vs. warm) on landing rates. We used two lure types to see whether the release-rig with small hooks had a consistent effect, and we controlled for water temperature because previous research in the Baltic has shown it affects deep hooking (Stålhammer et al., 2014).

On a substantially smaller subset of fish targeted with hardbaits only by the first author, additional data on hook wounds, injury and bleeding was collected from the Baltic. It was decided at random when to collect these data, and we confined this data collection only to the first author to minimize subjectivity effects caused by different people judging wounds. Unhooking time was defined as the number of seconds from the time of holding the fish ready to unhook until the hook was removed. We used a special hand-hold technique using the pike’s operculum—also called the “gill-grip”—to avoid delay in unhooking from hooks tangling in nets. When the hooks could not be grasped with the fingers, hooks were removed with forceps on the smaller hooks and more powerful pliers on the larger hooks. On critically and deeply hooked fish, e.g., in gills, hooks were cut off with a plier.

Hook wounds were estimated as an ordinal index: 1 = no visible wound, 2 = small to medium wound up to 2–3 mm wide/long, and 3 ≥ 3 mm wounds. Degree of bleeding was also enumerated as an ordinal bleeding index: 1 = no visible bleeding, 2 = small to medium bleeding with a small patch of blood, and 3 = heavy bleeding, blood pulsing out. If hooked on more than one hook, the degree of injury was defined as the injury from the hook causing most damage.

### Statistical analyses

The probability of landing was modelled using binary logistic regression with the predictor variables lure type (softbait vs. hardbait), hook mount (normal-rig vs. release-rig) and temperature (cool vs. warm). The injury outcomes (unhooking time, hook wound index, bleeding index) were analysed using a general linear model (GLM) assuming the three level ordinal index as a metric variable, using the hook mount, type of lure and water temperature as fixed effects. The significance level was set at *p* < 0.05, and the analyses were conducted with the SPSS software version 9.0 for Windows (SPSS, Inc., Chicago, IL, USA). In the case of non-significant co-variates (lure type and water temperature), the results were plotted in a pooled fashion by hook mount (normal-rig vs. release-rig) to aid in visual interpretation of the results.

## Results

**Figure 4:**
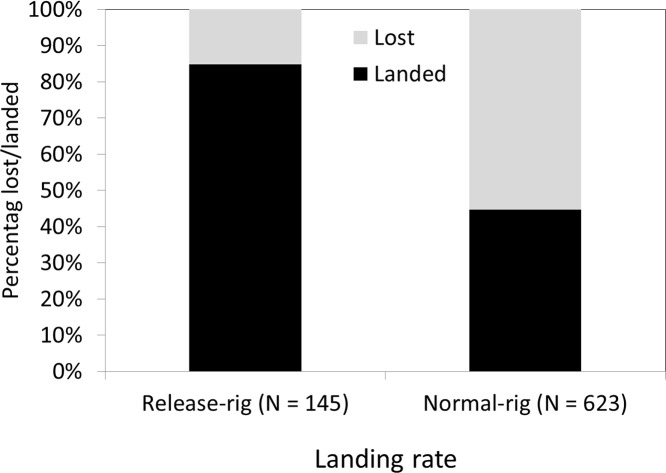
Landing rates in northern pike targeted with a normal-rig with large hooks and a release-rig with small hooks pooled across both lure types from Fig. 1. Statistical analyses in Table 1 show that only the release-rig was significant.

The pooled landing rate of traditionally mounted soft- and hardbaits was 44.7% (*N* = 623) and 84.9% (*N* = 145) for the same lures fished with release-rigs (Fig. 4). The logistic regression model revealed a highly significant, positive effect of the release-rig on landing rate, but the landing rates were independent of water temperature and lure type (Table 1).

**Figure 5:**
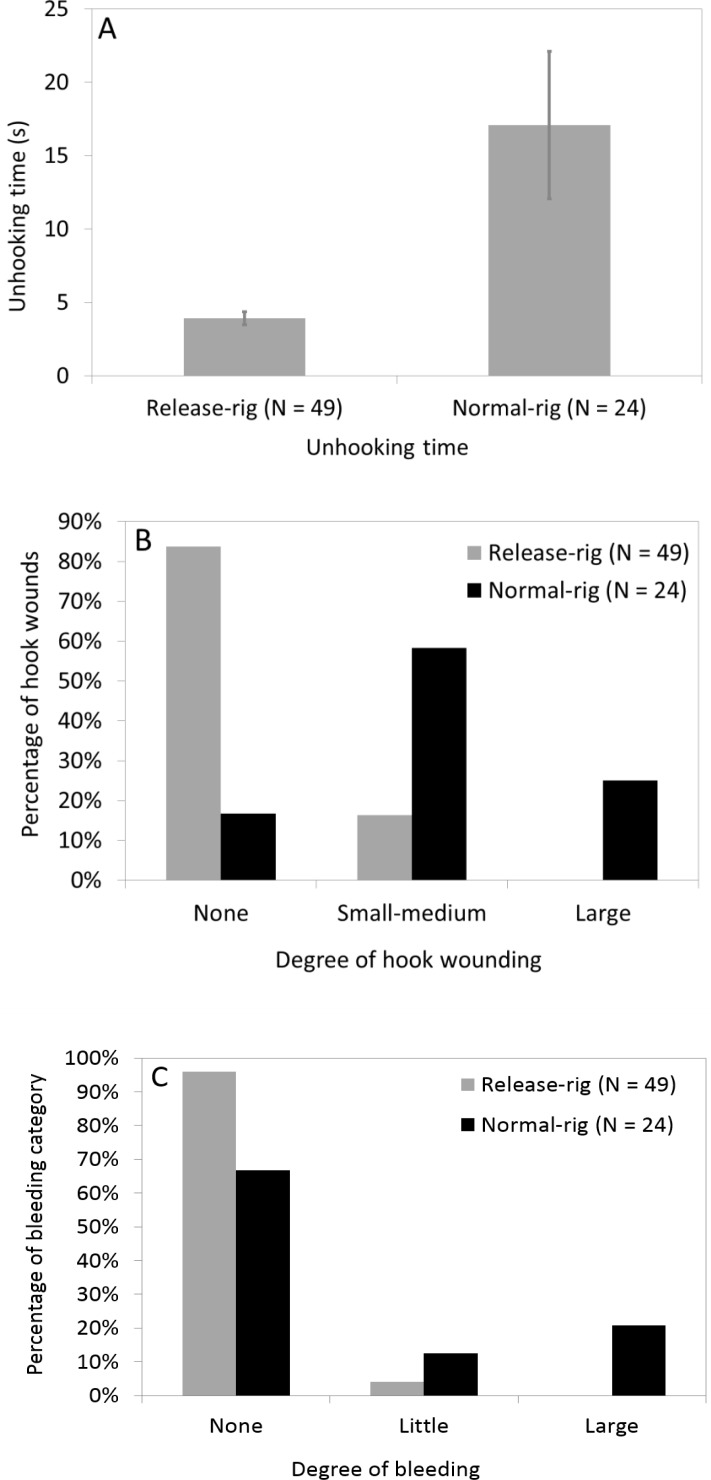
Unhooking time and injury with normal rig and release-rig. (A) Average unhooking time (in s, ±standard error, SE) in northern pike targeted with a normal-rig with large hooks and a release-rig with small hooks when fished with hardbait (Fig. 1B). Statistical analyses in Table 2 show that only the release rig was significant. (B) Degree of hook wound in three categories in northern pike targeted with a normal-rig with large hooks and a release-rig with small hooks fished with a hardbait (Fig. 2B). Statistical analyses in Table 2 show that only the release rig was significant. (C) Degree of bleeding in three categories in northern pike targeted with a normal-rig with large hooks and a release-rig with small hooks fished with hardbait (Fig. 2B). Statistical analyses in Table 2 show that only the release rig was significant.

The average unhooking duration was 17 s (*N* = 24) for traditionally mounted hardbaits compared to an average of 4 s (*N* = 49) for the same lure fished with release-rigs (Fig. 5A), and these differences were highly significant and unrelated to water temperature (Table 2). Relatedly, we found significant differences in the average hook wound index among normal-rig pike (*N* = 24) and pike captured with the release-rig (*N* = 49), with no effect attributed to water temperature (Table 2). 17% of all normal-rig fished pike lacked visible hook wounds, while a quarter of these had large hook wounds (Fig. 5B). By contrast, 84% of pike caught on release-rig lacked visible hook wounds, and no release-rig pike showed large wounds (Fig. 5B).

**Table 1:** Logistic regression of release-rig, lure type and water temperature on landing rate in Baltic northern pike (*N* = 768 cases). The raw data are plotted in Fig. 4.

Factor	*β* ± SE	*e*^*β*^ (odds ratio)	95% CI for *e*^*β*^ (odds ratio)	*P*-value	–
Lure type	0.0606 ± 0.818	10.625	0.9051–1.2473	0.4587	
Water temperature	−0.1005 ± 0.1548	0.9043	0.6677–1.2249	0.5160	
Release rig	1.9232 ± 0.2456	68.427	4.2286–11.0729	<0.001	
Constant	−0.2931 ± 0.3723			0.431	
Model					Chi^2^= 84.223, *df* = 3, *p* < 0.001, 61% correctly classified cases

Fish captured with normal (*N* = 24) or release-rig (*N* = 49) significantly differed in bleeding propensity (Table 2), with water temperature being again not significant. When fishing the normal hardbaits, two thirds of the pike had no bleeding and one fifth showed heavy bleeding, while the overwhelming majority of 96% of release-rig captured fish did not bleed at all, and no release-rig fish bled heavily (Fig. 5C).

**Table 2:** General linear models testing the impact of release-rig and water temperature during capture on unhooking time, hook wounding index and bleeding index in Baltic northern pike. The raw data are plotted in Fig. 5.

Factor	*F*-value	*P*-value
*Unhookingtime (s)*		
Intercept	23.349	<0,001
Temperature	0.119	0.731
Release rig	12.475	0.001
Model	*F* = 6.881	*P* = 0.002
	Adjusted *R*^2^ = 0.140	
*Hook wounding index*		
Intercept	628.918	<0.001
Temperature	0.190	0.664
Release rig	53.822	<0.001
Model	*F* = 29.053	*p* < 0.001
	Adjusted *R*^2^ = 0.438	
*Bleeding index*		
Intercept	364.242	<0.001
Temperature	0.607	0.493
Release rig	13.931	<0.001
Model	*F* = 8.284	*p* = 0.001
	Adjusted *R*^2^ = 0.168	

## Discussion

Using observational data collected using a citizen science approach with avid pike anglers we showed that the landing rates of northern pike almost doubled with the use of the release-rig compared to the same lure types fished with traditional hook mounts. Such result is very likely of large interest to anglers interested in maximizing their catch. We also found the injury and injury-related indicators previously associated with hooking mortality in pike, in particular bleeding (Arlinghaus et al., 2008a), to be reduced by the release-rig compared to the normal-rig. This suggests the use of the release-rig may also benefit those fish that are released after capture. Our data generally suggests a combined effect of two modifications related to the hook mount and hook size as represented in the release rig on landing rate and injury, but our design does not allow inferring about the relative effects of the hook mount and the hook size as we lack observations on traditionally mounted lures with small hooks or release-rigs with large hooks. It was not our aim to present a full factorial design. Instead, our aim was to study the practical outcome of a possible gear innovation as a combined effect of altered hook mounts facilitating smaller hook sizes on artificial lures to serve as a proof-of-concept and to examine whether citizen scientists would be useful data collectors in gear effectiveness studies in angling. We suggest conducting further experimental work to generate cause-and-effect inferences at a high level of mechanistic detail, in particular in relation to the relative effect of hook mount and hook size in release-rigs. Until such research becomes available, the combined effect of jointly altering the hook mount and reducing the hook sizes suggests the potential for release-rigs elevating landing rates and reducing fish injury. These aspects are of large practical relevance.

In-line hook mounts have existed in recreational fishing for decades, but the innovation of release-rigs far extends this principle and mainly relates to its ability to use much smaller hooks compared to traditional hook mounts—including in-line and line-thru systems. There are three main reasons why much smaller hooks than the standard must be mounted differently to work well in practical fishing situations. First, in small, short hooks with more narrow hook gapes mounted normally, the hook points will not extend much from the lure and therefore stands a lower chance of getting a grip in the fish mouth compared to larger hooks extends further out from the lure. Second, small hooks mounted normally on hardbaits stands a higher risk of collapse or being teared out in fight with a big fish if leverage from the lure to the hook multiplies the pressure on the hook. And finally, the weight from the lure hurled around the head of the fish during fight can act negatively on the hook hold on both hard- and softbaits, especially if small hooks are combined with heavy baits. The release-rig address all three issues: first, the rig runs parallel with the lure in its full length 1–2 cm away from the bait, creating a better exposure of the small hooks. Second, the rig is semi-fixed in the rear end and sliding in just one point in the front, fully eliminating the leverage from the lure. Third, when hooked the rig is released from the lure, so it can slide up the trace, minimizing negative effects on the hookhold caused from the weight of the lure hurling around. This stands in contrast to traditional “in-line” or “line-thru” systems, which only fully address the third point and only partly the second point. The reason for the latter is that leverage can arise easily from the critical moment between hitting the lure and the first shake of the head and it can also happen in the periods of the fight where the lure is bouncing back on the hook. Such effects also apply to soft in-line swimbaits because the in-line channel runs through a stiff, weighted front part of the lure. By contrast, in the release-rig no such effects are possible. Another aspect of how release-rigs differ from in-line rigs is that all types of baits/lures can be used or modified to be used with release-rigs or variants. This means that all anglers can use the baits/lures they already have, without having to buy a specific new lure. By contrast, rigs made for in-liners can only be used on the very same in-line lures, limiting the use to anglers having the possibility to buy the specific in-line lures.

Previous studies in pike have shown that smaller lures/baits are inhaled deeper, with a greater risk of gill-hooking being closely linked to more bleeding and higher mortality (Arlinghaus et al., 2008a). In our study, the ingestion of large (standard rig) or small hook sizes (release rig) was not constrained by physical gape limits as we standardized the lure sizes. Thus, any injury-related effect related to the hook mounting cannot have been caused by physical hook size-gape limits. Indeed, while controlling for lure size and type, we found release-rigged lures fished with smaller hook sizes to increase landing rates and reduce injury compared to normal-rigs with larger hooks. Similarly, Rapp, Cooke & Arlinghaus (2008) showed that a reduction in hook sizes when bottom fishing with natural bait (corn) not only increased landing and catch rates but also size of carp, suggesting that the smaller hook size improved retention on the gear. In the study of Rapp, Cooke & Arlinghaus (2008), the improved landing and catch rates with the smaller hooks was not explained by better penetration and hook hold, which we prefer as explanations (see video and Fig. 3), but by the specific feeding pattern of the carp sucking in the bait from the bottom. The authors speculated that smaller hooks were lighter and therefore more easily taken up by the carp, resulting in a better chance of hooking the fish. We argue instead that smaller hooks penetrate more easily and produce a better hook hold, particularly in top predators with bony jaws (Fig. 3). Lures fished on release-rigs were added extra weight (power-dots) on the belly of hardbaits—and so-called nail-weights in the softbaits—to compensate for the smaller and lighter hooks on the release-rig. Consequently, there were no weight differences on lures of the same size used for spinning pike with and without release-rigs, and thus possible weight differences were controlled. Moreover, as the release-rigs hang a bit under and away from the lure the three dimensional space taken up by lure, rig and smaller hooks on a release-rigged bait was the same as on the original lures with much larger hooks mounted directly on the lure itself. Lure size rather than the specific hook mount controls the sizes of pike that are captured (Arlinghaus et al., 2008a). Although we did not record fish sizes, we therefore consider it unlikely that release-rigged lures will be inhaled more deeply compared to same-sized lures mounted normally. The majority of pike caught in this study were 50–80 cm with fish up to 110 cm captured during the study, which is in agreement with previous work in pike that reported that sublegal pike below 50 cm were unlikely to be captured when lure sizes were 15 cm or larger (Arlinghaus et al., 2008a).

Although we prefer the explanation that our release-rig effects were mainly caused by the smaller hook size, our design confounded hook size and hook mount. Thus, our explanation to explain the combined effect we report remains speculative. As mentioned before, further work is needed to disentangle the relative importance of hook size and hook mount in explaining landing rates and injury in pike and other predators.

One limitation of our work is that the normal and release rig data were collected subsequently and that the volunteer anglers were free to choose when to use a particular lure type on any given day. It is our experience that keeping the motivation high in citizen science projects with anglers demands to allow anglers some level of free choice. This is at the cost of losing scientific control about possible confounding factors. In our study, we thus cannot discount the possibility that year and lure type timing effects confounded the results, but we think these effects are unlikely and if present did not strongly affect the results. There were not obvious changes in the Baltic Sea during the short period of time when the observational data was collected. The anglers that collected the data in the two periods have remained the same, and we failed to find a temperature effect on landing rates and injury. Annual temperature is the most decisive factor that might systematically differ from year to year and is known to affect catch rates in pike (Kuparinen, Klefoth & Arlinghaus, 2010), but this effect was controlled in our work. Further work is nevertheless needed following an experimental design, and data on fight time and size of fish will be important to collect. This is also the reason why we present a substantially smaller sample size for the injury data compared to the landing rate data as we choose to collect these data at a high quality in a standardized fashion through the first author only. The uncertainty remains that uncontrolled variables affected the study outcomes, which is why we consider our results to constitute preliminary findings and a proof-of-concept that warrants a fully controlled experimental study to substantiate the results we present.

## Conclusions and Implications

Our work presents a new type of rigging for lure angling in predators that is suggestive of increasing landing rates and reducing injury. Adopting the rig thus promises to help both anglers and fish. Given its higher landing rate, the release-rig may indeed meet good acceptance by predator anglers. The outlook for the fish is more mixed. On the one hand, the release-rig may land a greater fraction of fish that attack the lure. On the other hand, the fish that are to be released may be in better shape compared to the same fish being captured with normal-rigs. Moreover, the release-rig we designed strongly reduced unhooking time, which reduces the likelihood of inadvertent wounding during handling. Further fully controlled experimental studies are needed to disentangle cause and effect and to see whether the data presented here for northern pike fished in the Baltic can be recovered under fully controlled settings and also holds true for other piscivorous fish species.

##  Supplemental Information

10.7717/peerj.4744/supp-1 Data S1 Raw data

## Additional Information and Declarations

### Data Availability

The following information was supplied regarding data availability: The raw data are provided at Figshare: https://doi.org/10.6084/m9.figshare.6198368. A video describing the functionality of the release-rig is available at https://doi.org/10.6084/m9.figshare.5833803.
